# Elaboration of a nursing record standard for an Emergency Care Unit^*^

**DOI:** 10.1590/1980-220X-REEUSP-2022-0253en

**Published:** 2023-05-29

**Authors:** Dilzilene Cunha Sivirino Farias, Eliane de Fátima Almeida Lima, Karla de Melo Batista, Marcia Regina Cubas, Júlia Valéria de Oliveira Vargas Bitencourt, Cândida Caniçali Primo

**Affiliations:** 1 Universidade Federal do Espírito Santo, Vitória, ES. Brazil.; 2 Pontifícia Universidade Católica do Paraná, Curitiba, PR. Brazil.; 3 Universidade Federal da Fronteira Sul, Santa Catarina, Brazil.

**Keywords:** Emergencies, Nursing Diagnosis, Electronic Health Records, Standardized Nursing Terminology, Nursing Theory, Urgencias Médicas, Diagnóstico de Enfermería, Registros Electrónicos de Salud, Terminología Normalizada de Enfermería, Teoría de Enfermería, Emergências, Diagnóstico de Enfermagem, Registros Eletrônicos de Saúde, Terminologia Padronizada em Enfermagem, Teoria de Enfermagem

## Abstract

**Objective:**

To develop a registration standard with diagnoses, outcomes and nursing interventions for an Emergency Care Unit.

**Method:**

This is applied research of technological development developed in three steps: elaboration of diagnoses/outcomes and interventions statements following the International Classification for Nursing Practice; assessment of diagnosis/outcome relevance; organization of diagnosis/outcome and interventions statements according to health needs described in TIPESC.

**Results:**

A total of 185 diagnoses were prepared, of which 124 (67%) were constant in the classification, and 61 had no correspondence. Of the 185 diagnoses, 143 (77%) were rated as relevant by 32 experienced emergency room nurses, and 495 nursing interventions were correlated to diagnoses/outcomes.

**Conclusion:**

It was possible to build a record standard for the Emergency Care Unit following standardized terminology, containing diagnostic statements/outcomes and relevant interventions for nursing practice assessed by nurses with practice in emergency.

## RESUMO

**Objetivo:**

Elaborar um padrão de registro com diagnósticos, resultados e intervenções de enfermagem para Unidade de Pronto Atendimento.

**Método:**

Pesquisa aplicada de desenvolvimento tecnológico, desenvolvida em três etapas: elaboração dos enunciados diagnósticos/resultados e intervenções seguindo a Classificação Internacional para Prática de Enfermagem; avaliação da relevância dos diagnósticos/resultados; organização dos enunciados diagnóstico/resultado e intervenções, conforme necessidades de saúde descritas na TIPESC.

**Resultados:**

Foram elaborados 185 diagnósticos, dos quais 124 (67%) eram constantes na classificação, e 61 não tinham correspondência. Dos 185 diagnósticos, 143 (77%) foram avaliados como relevantes por 32 enfermeiros experientes em urgência e emergência, e 495 intervenções de enfermagem foram correlacionadas aos diagnósticos/resultados.

**Conclusão:**

Foi possível construir um padrão de registro para Unidade de Pronto Atendimento seguindo terminologia padronizada, contendo enunciados diagnósticos/resultado e intervenções relevantes para prática de enfermagem avaliada por enfermeiros com prática em urgência e emergência.

## RESUMEN

**Objetivo:**

Elaborar un patrón de registro con diagnósticos, resultados e intervenciones de enfermería para la Unidad de Atención de Emergencia.

**Método:**

Investigación aplicada de desarrollo tecnológico, desarrollada en tres etapas: elaboración de declaraciones de diagnósticos/resultados e intervenciones siguiendo la Clasificación Internacional para la Práctica de Enfermería; evaluación de la relevancia de los diagnósticos/resultados; organización de las declaraciones diagnóstico/resultado e intervenciones, de acuerdo con las necesidades de salud descritas en TIPESC.

**Resultados:**

Se elaboraron 185 diagnósticos, de los cuales 124 (67%) fueron constantes en la clasificación y 61 no correspondieron. De los 185 diagnósticos, 143 (77%) fueron evaluados como relevantes por 32 enfermeros experimentados en urgencias y emergencias, y 495 intervenciones de enfermería fueron correlacionadas con los diagnósticos/resultados.

**Conclusión:**

Fue posible construir un estándar de registro para la Unidad de Atención de Emergencia siguiendo una terminología estandarizada, que contiene declaraciones de diagnóstico/resultado e intervenciones relevantes para la práctica de enfermería evaluada por enfermeros con práctica en urgencia y emergencia.

## INTRODUCTION

Using standardized nursing language systems for electronic health records has been identified as an important tool to improve the quality of nursing care documentation and generate safe and reliable information for continuity of care^(1,2)^.

Among the language systems, the International Classification for Nursing Practice (ICNP^®^) allows the composition of nursing diagnoses, outcomes and interventions that can be inserted into computerized systems, collaborating to demonstrate the mastery of nursing practice worldwide^(2,3,4)^.

This terminology has been in a continuous process of development by the International Council of Nurses (ICN) since the 1990s, with the aim of unifying nursing language, enabling greater scientific advancement of the profession as well as allowing this standardized language to be incorporated into health information and communication systems^(5)^.

Thus, to make it more accessible and applicable to clinical practice, the ICN has encouraged the construction of a terminological subset or ICNP^®^ catalog. These are considered essential technological instruments for nursing practice in a specific context, reflecting individualized, systematized and humanized care^(6)^. In Brazil, researchers have used part of the steps in the terminology subset elaboration guideline to develop a recording standard for nursing evolution with specialized nursing terms based on this terminology, in order to facilitate the records and encourage the incorporation of the Nursing Process steps in clinical practice^(7,8)^.

The Nursing Process is a methodological instrument by which nurses think and perform care, requiring cognitive, affective and operational skills for its application^(9)^. The use of this methodology in professional practice takes place through five dynamic and interrelated steps, such as data collection, nursing diagnosis, nursing planning, implementation and nursing assessment, as diagnosis and prescription of nursing actions are nurses’ private activities^(10)^. All these steps must be recorded by professionals in a systematic, orderly and understandable way, regardless of the format of the medical record, whether traditional or electronic^(11)^.

If, on the one hand, the lack of systematic and standardized registration leads to loss of relevant patient information, makes continuity of care unfeasible and makes it difficult to carry out research for the development of the profession, on the other hand, the institutionalization of instruments with standardized language can improve nursing record quality scores, bringing safety to care, assisting in research that creates predictive models and demonstrating the contribution of nursing to health care quality^(12,13)^.

However, despite the recognition of the importance of recording in a systematic and standardized way in different scenarios, many professionals and services find it difficult to document their actions with a language specific to nursing^(14)^. In emergency services, because it is a dynamic assistance with quick response, high turnover of patients and overload of administrative activities, nurses have difficulty planning assistance following all Nursing Process steps and registering them in patients’ records^(2,15)^.

In view of this, there is a need to seek strategies to overcome these challenges, creating mechanisms to facilitate the application of the Nursing Process as a care tool as well as ensuring the recording of all its steps with a standardized language^(12–16)^. However, for the Nursing Process not to be an end in itself, representing only a method of grouping information, it is necessary to be based on Nursing Theories. This theoretical support must express the philosophical view of the nursing care model that is intended to be provided, depending on the field of action and life conceptions and the world that the nursing group has^(17)^.

The Theory of Praxis Intervention in Collective Health Nursing (TIPESC – *Teoria de Intervenção Práxica da Enfermagem em Saúde Coletiva*), derived from collective health, with a philosophical basis in historical and dialectical materialism, is a framework that can be used to organize nursing care in different scenarios, as it brings expanded health-disease process and human needs conceptions. It presents the needs that are distinguished in natural or life preservation, properly human needs and alienated needs. Its characteristics encourage professionals to develop nursing care in a reflective and critical way, considering the social, political, cultural context, converging with the principles and guidelines of the health model adopted in the country^(18,19)^.

In this regard, this research aimed to elaborate a registration standard with diagnoses, outcomes and nursing interventions for an Emergency Care Unit.

## METHOD

### Study Design

This is applied technological development research carried out between January 2019 and May 2021, which followed three steps: elaboration of diagnoses/outcomes and interventions statements following the International Classification for Nursing Practice; assessment of diagnosis/outcome relevance; organization of diagnosis/outcome and interventions statements according to health needs described in TIPESC^(19,20)^. This article was structured following the criteria established in the Standards for Reporting Qualitative Research: a synthesis of recommendations (SRQR).

### Place, Population and Selection Criteria

The research was carried out in the city of Vitória, located in the metropolitan region of Espírito Santo, Brazil. Nurses from the metropolitan region of Espírito Santo participated in content assessment. For the composition of experts, nurses with a minimum academic degree of expert and experience of at least two years in direct patient care in emergency units were included. Professionals who were on leave, on vacation or on extended medical leave (more than 30 days) were excluded.

### Data Collection

In the first step for elaborating the diagnostic statements, a literature review was carried out with a search for articles in the Medical Literature Analysis and Retrieval System Online (MEDLINE), Latin American and Caribbean Literature on Health Sciences Information (LILACS), Cumulative Index to Nursing and Allied Health Literature (CINAHL) and Nursing Database (BDENF) databases. Articles indexed in CINAHL were accessed through the CAPES portal, and the search for publications took place through the Virtual Health Library (VHL), Scientific Electronic Library Online (SciELO) virtual repository and PubMed with the descriptors “Nursing Diagnosis”, “Emergency Nursing”. Study selection took place from June to August 2019, and consisted of 21 articles.

Then, a survey of the Manchester Triage System^®^ flowcharts and discriminators was carried out from 160,604 risk stratification recorded by nurses from two Emergency Care Units in the metropolitan region of Espírito Santo from January to June 2019. The identified terms were submitted to electronic cross-mapping with the Access^®^ software, version 2016, and manual mapping with ICNP^®^ 2019 focus axis, following the steps described in ISO/TR 12300:2016^(21)^. In this process, constant and non-constant terms were identified. Regarding non-constant data, these were independently analyzed by the main researcher and a nurse, a master’s student specializing in auditing and medical bills.

For the elaboration of diagnostic statements/outcomes, the terms resulting from the review and mappings were used following the ICN and ISO 18.104:2014 guidelines^(22)^. Moreover, the ICNP^®^ 2019 pre-coordinated statements, the Risk Stratification System concepts, scientific articles and gray literature referring to emergency situations were used as an empirical basis. Operational definitions of nursing diagnoses/outcomes were not constructed, considering that they were not part of the scope of this project due to the time required by this step, and will be addressed in a future study.

Interventions were related to the elaborated nursing diagnoses/outcomes, considering the pre-coordinated ones in ICNP^®^, those available in the Nursing Interventions Classification (NIC) and the interventions identified in the literature. The last two situations underwent a process of adaptation of terms with ICNP^®^ 2019, considering the ISO 18.104:2014^(22)^ guidelines on nursing intervention elaboration.

In the second step, to assess diagnostic statements/outcomes, an invitation letter to participate via WhatsApp^®^ groups was sent to 120 nurses who work in an Emergency Care Unit in the metropolitan region of Espírito Santo. The disclosure in the groups took place through the researcher’s relational network and the nursing coordination services. Three distinct groups of evaluators were arranged: group 01 – nurses from the Emergency Care Unit in São Pedro; group 02 – nurses from the Emergency Care Unit in Praia do Suá; and group 03 – nurses from the Emergency Care Units in Cariacica, Vila Velha and Serra. Each group assessed a certain set of statements, which was available for assessment in an instrument prepared in Google Drive Forms for 45 days.

The instrument for data collection was structured and divided into three sections. The first section presented the study and objectives to nurses and sent them to read the Informed Consent Form (ICF) to accept or reject participation. In the second section, respondents had access to online filling in the data referring to expert characterization, and the third section contained a list with diagnostic statements/outcomes for assessment on a Likert-type scale, containing the following options: 1 – Totally adequate; 2 – Adequate; 3 – Little adequate; 4 – Inadequate.

Experts assessed the relevance/occurrence of diagnostic statements/outcomes for emergency according to their practical experience, clicking on the option they judged most appropriate. There were also spaces for comments and suggestions, if deemed necessary.

### Data Analysis

Data were analyzed using the Content Validity Index (CVI), which measures the agreement among experts on certain aspects of the instrument and its items^(23)^. Comments and suggestions were checked against the literature in the area.

The CVI calculation was the sum of the agreement of answers marked “1” (Completely adequate) and “2” (Adequate) divided by the total number of answers given by experts.

Items with CVI ≥ 0.80% were considered validated, and diagnoses/outcomes that showed lower agreement were disregarded^(8)^.

In the first round of assessment of the 120 invited experts, 32 nurses agreed to participate and met the inclusion criteria. Nurses were divided into three groups: groups 01 and 03 – composed of 11 professionals and group 2 – 10 nurses. In the second round, the utterances that did not obtain a CVI ≥ 0.80% were submitted to a new assessment. It was forwarded to the 32 initial respondents, via email, link to access the new assessment instrument, containing the statements diagnosis/non-validated outcomes, and 22 nurses, out of the 32 guests, answered the assessment instrument.

### Ethical Aspects

The study was approved by the Research Ethics Committee of the Health Sciences Center of the *Universidade Federal do Espírito Santo*, under Opinion 3,765,064, 2019. All ethical and legal requirements governing research with human beings established by Resolution 466/2012 of the National Health Council were respected, with the signature of ICF by participants.

## RESULTS

In the first step, based on the analysis of the 21 articles after literature review, 85 diagnoses from NANDA International and 33 from ICNP^®^ were identified. In the survey of flowcharts and discriminators, 114 terms relevant to nursing practice were identified and mapped. From these bases, it was possible to elaborate 185 statements, of which 124 were constant, and 61 were not found to correspond to ICNP^®^ 2019.

As for the profile of evaluators, of the 32 experts, 50% between 41 and 50 years old; 43.75% were between 35 and 40 years old; and 6.25% were over 50 years old. The majority 25 (78.13%) were female. The mean training was 17 years, and the maximum title was master, with 03 respondents and 29 were experts. The mean time working in the emergency service was 11 years. Of these, 15 nurses worked between 1 and 5 years in the current workplace, 12, between 6 and 10 years, and 5 nurses worked for more than 11 years in the same service.

Regarding assessment, in the first round, 108 diagnoses obtained CVI ≥ 0.80%. In the second round, of the 77 statements assessed, 35 obtained a CVI ≥ 0.80%. Thus, adding the first and second rounds, of the 185 diagnoses/outcomes assessed, 143 (77.3%) statements had a CVI ≥ 0.80% (Chart 1) and 42 (22.7%) were not validated.

**Figure 1:**
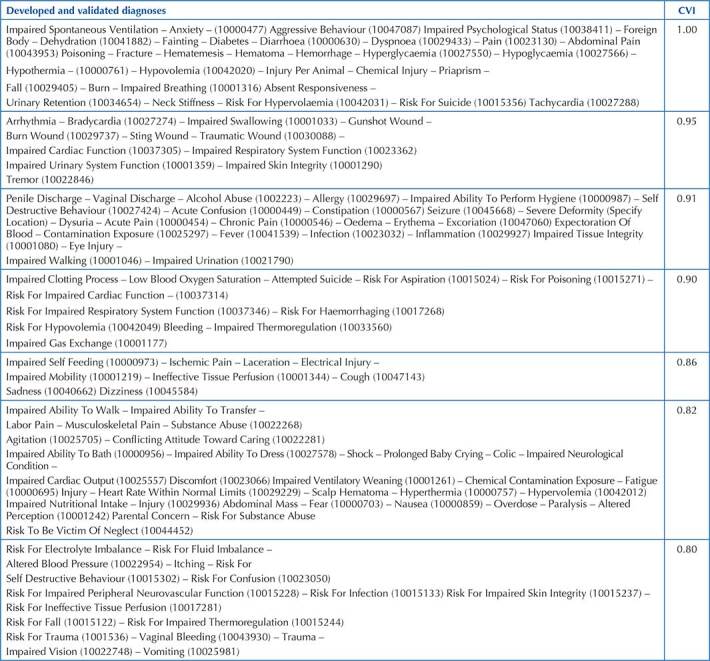
List of diagnostic statements/outcomes with the respective ICNP^®^ code arranged according to the Content Validity Index – Vitória, ES, Brazil, 2021.

Source: author, 2021.

Diagnoses were identified in two categories of needs: natural needs and properly human needs (Chart 2). Natural needs obtained the largest grouping of statements, with emphasis on regulation, safety and integrity with 56, 26 and 24 listed diagnoses/outcomes, respectively. Diagnostics for alienated needs were not validated.

**Figure 2:**
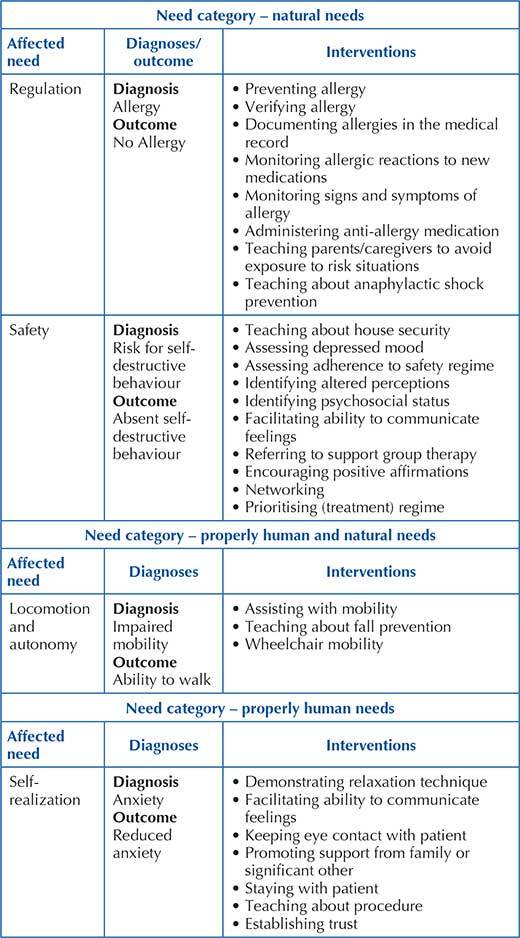
Breakdown of the distribution of diagnoses/outcomes and interventions according to the need category – Vitória, ES, Brazil, 2021.

Source: author, 2021.

Terms belonging to two categories were identified simultaneously, such as diagnosis Gunshot Wound, which has the need for integrity (properly human need) and safety (natural need) affected, and diagnosis Impaired Mobility, with the need to locomotion (natural need) and autonomy need (properly human need) compromised.

From the diagnoses statements, ICNP^®^ 2019 pre-arranged nursing interventions were related, identified in the NIC and in the literature review that were applied to emergency clinical situations. After a process of standardization of terms and cross-mapping with pre-combined ICNP^®^ interventions, it was possible to list 605 interventions, 308 of which are constant and 297 are new interventions.

Diagnoses that did not reach CVI ≥ 0.80% were excluded and 110 interventions that comprised these diagnoses were excluded. It is clarified that, for this study, nursing interventions were not submitted to experts’ assessment, since, as they were extracted from ICNP^®^ 2019 and NIC, they were already submitted to an assessment process by their expert committees.

In the end, the registration pattern was composed of 143 nursing diagnoses/outcomes and 495 nursing interventions divided into 16 needs within two need categories presented at TIPESC^(19)^. Considering the number of pages to present all diagnoses/outcomes and interventions, they are available for consultation at the link: https://enfermagem.vitoria.ufes.br/ptbr/posgraduacao/PENF/disserta%C3%A7%C3%B5es-defendidas.

## DISCUSSION

The adoption of a standardized and specific nursing language to describe their clinical practice results in clearer and more objective records, facilitating the communication process and promoting greater visibility of their work^(12–16)^. Thus, the elaboration of a registry standard with diagnoses assessed by expert nurses with experience in care can demonstrate their relevance in clinical practice.

In this study, the profile of experts with experienced professionals in emergency and low academic training at the *stricto sensu* level predominated. Expert selection for validity studies has been identified as a critical issue, both in defining the profile and in attracting and guaranteeing participation^(23)^.

A study identified that expert nurses’ judgment was positively influenced by factors such as age, time working as a nurse in the area studied for certain defining characteristics; meanwhile, for others, the completion or not of a graduate course on Nursing Process or diagnosis, use or not of the taxonomy in professional practice and experience in nursing diagnosis influenced the judgment^(24)^.

This fact demonstrates the need for a balance between professional experience and academic training when selecting professionals who will be part of the panel of evaluators^(23)^.

In the construction process of nursing statements/diagnoses, it was possible to identify a large number of terms contained in ICNP^®^ 2019, demonstrating the capacity that this terminology has to represent professional practice in different scenarios^(6–8)^. On the other hand, with the assessment of emergency experts, it was possible to perceive that some terms are not constant in this version, such as Foreign Body, Penile Discharge, Vaginal Discharge, Severe Deformity, Priapism, Neck Stiffness, Abdominal Mass, were considered relevant for care.

Associated with this, it was verified that, of the 61 non-constant diagnoses, 30 statements were assessed as relevant for practice. Research carried out in a hospital specialized in emergency and trauma mapped 1,431 nursing records with a degree of equivalence with ICNP^®^ 2013 terms, and identified 63 new terms, i.e., not constant^(25)^. This indicates that although ICNP^®^ is comprehensive, it is not sufficient to represent practice in specific contexts, requiring systematic use of studies in this area, as recommended by ICN^(3,5)^.

Using the need category described in TIPESC^(20)^ allowed us to identify that most of the elaborated and validated diagnosis/outcomes statements are in the natural need category, i.e., those related to life maintenance. This finding is totally predictable, since in emergency care interventions to restore patients’ vital functions are a priority. However, even during the stabilization process, even if severity persists, it is necessary to analyze the health phenomenon in its various facets and meet all individuals’ needs^(25–27)^, for which the reach of these results in attention to other dimensions of care that can promote health or illness; therefore, from a perspective effectively expanded to human health.

In this regard, to identify nursing diagnoses according to the health need category presented in TIPESC, whose theoretical and philosophical basis is based on the principles of collective health and historical and dialectical materialism in a scenario with a predominance of development of care guided by signs and symptoms of a strictly biological nature, it tends to introduce the materialization in practice of a model of care that pays attention to the other dimensions of care. A theoretical framework in the field of contemporary nursing that raises in professionals a critical and reflective view of the social group to which they belong, identifying the contradictions that are present in the health-disease process, created a perspective of care that goes beyond a momentary clinical stabilization. It seeks to employ actions that cause transformations, overcoming the contradictions present in the daily routine of health care and nursing in the face of the community^(19)^.

Therefore, with the organization of a nursing record standard in emergency services supported by the vision of needs identified in TIPESC, the genesis of a transformation of existing practices is envisioned, instigating professionals to develop nursing care in a reflective and critical way, considering the social, political, cultural contexts, converging with the principles and guidelines of the health model adopted in Brazil^(18,19)^.

Another relevant aspect was the considerable amount of risk diagnostic statements/outcome 30 (16.2%) of which 66% were validated. It drew attention in this study that diagnoses Risk To Be Victim of Child Abuse, Risk To Be Victim of Elder Abuse, Risk To Be Victim of Intimate Partner Violence did not reach consensus among nurses as relevant for emergency care. This may demonstrate how nursing care is still influenced by a disease-centered biomedical model, in which an isolated, punctual intervention prevails, with the objective of immediate cure^(26,27)^.

Such findings reinforce that systematized nursing care, anchored in a theory, is essential for actions to be organized in order to identify and treat each individual as a whole, directing care to the unique needs that are historically and socially constructed^(18)^.

Perceiving cases of intrafamily violence in the emergency context is complex, as it involves several barriers that make it difficult to deepen the investigation, among which are: emphasis on flow; professionals’ work overload; disarticulation with reference services; professionals’ frustration due to their inability to solve the problem or help; isolation of emergency teams; and lack of professional training. However, many victims of violence have their first contact with this service, and professionals must be aware of signs and symptoms, such as fractures and bruises, lacerations and traumas, palpitations, shortness of breath and chronic pain^(27–29)^.

Intrafamily and community violence affect people unequally. Children, adolescents, women (of all ages) and older adults are the groups most affected by intrafamily violence, and it is estimated that one in three female patients are victims of intimate partner violence^(28)^. Therefore, recognizing that a fracture can be a related need in the natural need category of physical integrity, conservation of life, but that can also be the result of social inequalities that portray vulnerable social groups’ needs (older adults, women, children), i.e., historically constructed needs (freedom), allows nurses to plan and carry out interventions considering the contradictions and the search for their overcoming.

The large percentage of diagnosis related to pain was also highlighted. Corroborating with research carried out in an emergency unit in southern Brazil, which identified pain as the main nursing diagnosis of patients classified by the Manchester protocol^(29)^.

Pain is a physiological phenomenon that affects a person’s physical integrity, reducing quality of life and causing pathophysiological changes that contribute to the emergence of organic and psychological comorbidities. An individual with pain can bring socioeconomic consequences similar to those caused by cardiovascular diseases^(30)^.

Managing pain in urgent and emergency services is part of nurses’ role, encompassing the process of identifying, assessing, diagnosing and promoting appropriate nursing interventions for patient pain, clinical status, history and preference characteristics. From the perspective of the needs registered in TIPESC, it is considered that pain is a phenomenon that may be in properly human and natural need categories, because, despite pain being a pathophysiological commitment, it affects individuals in different ways. Individuals with acute pain, who are formally inserted in the labor market, will seek to satisfy their needs differently from autonomous microentrepreneurs or large entrepreneurs. Thus, administering analgesics may not be the only intervention necessary to meet individuals’ needs.

As for the interventions listed in the registration pattern, it is estimated that they present actions that focus, for the most part, on the biological being’s recovery. This occurs because elaborating diagnoses starts from terms predominantly originating from a tool whose structure is essentially developed in the positivist view. Moreover, the publications found in these scenarios are also steeped in this worldview.

However, given the prerogatives of emergency services inserted in a public health policy that provides for health interventions that look at the community, with articulated, intersectoral actions, aimed at stabilizing the individual, but also at health promotion and prevention, it should be noted that they were associated with diagnoses, interventions that can be worked on from the perspective of the needs defended in TIPESC.

Such interventions can be implemented, guided by historical and dialectical materialism, to occur in a participatory and dynamic way, leading nursing care from broader concepts of the health-disease process and health needs^(18,19)^.

Thus, practice guided by contemporary nursing theory can help to improve quality of care, as it allows nurses to articulate what they do for patients and why. Therefore, they must continue to guide their practice through the lens of nursing theory, in addition to assessing the effectiveness of this practice guided by them^(17)^.

### Limitations

The limitation is the elaboration of diagnostic statements based on the Manchester Triage System^®^ flowcharts and discriminators, which privileges biological aspects as well as its validity occurred by professionals with a profile that maintains the focus of attention on biological needs. Another limitation refers to the lack of knowledge of Espírito Santo nurses regarding the chosen theoretical model, which may have compromised the non-validity of nursing diagnoses/outcomes in alienated needs.

## Conclusion

The registration standard prepared and validated by 32 expert nurses in emergency care contains 143 diagnostic statements/outcomes and 495 nursing interventions, following the ICNP^®^ terminology and organized by the needs described in TIPESC.

The organization of terms, according to the needs described in TIPESC, indicated the prevalence of diagnoses in natural needs, i.e., biological. The non-validity of diagnoses related to social issues points to the importance of professionals broadening their gaze to issues beyond physiological changes and breaking with the model centered on medical care.

It is believed that the elaboration of a registration standard, with a terminology that adapts well to computerized systems, containing diagnostic statements/outcomes assessed as relevant for emergency, is a useful technological tool to organize nursing care management, document care and highlight the actions of this team in these services, which are often invisible.
